# New-Onset Diabetes Mellitus in Post-renal Transplant Patients on Tacrolimus and Mycophenolate: A Systematic Review

**DOI:** 10.7759/cureus.31482

**Published:** 2022-11-14

**Authors:** Savitri Aninditha Nandula, Chinmayi Sree Boddepalli, Sai Dheeraj Gutlapalli, Vamsi Krishna Lavu, Rana Abdelwahab Mohamed Abdelwahab, Ruimin Huang, Shanthi Potla, Sushen Bhalla, Yousif AlQabandi, Prachi Balani

**Affiliations:** 1 Internal Medicine, California Institute of Behavioral Neurosciences & Psychology, Fairfield, USA; 2 Dermatology, California Institute of Behavioral Neurosciences & Psychology, Fairfield, USA; 3 Psychiatry, California Institute of Behavioral Neurosciences & Psychology, Fairfield, USA

**Keywords:** anti-hyperglycemic drugs, tacrolimus, immunosuppressant therapy, post-transplant diabetes mellitus, renal transplantation

## Abstract

A frequent complication in kidney transplantation is post-transplant diabetes mellitus (PTDM). The primary goal of this study is to review the risk factors and preventive methods and compare the different available anti-diabetic medications for the management of PTDM. We searched databases like Pubmed and Google Scholar for related articles using specific terms and phrases. Following a thorough investigation, we applied the inclusion and exclusion criteria and completed a quality assessment.

Modifiable risk factors have a significant role in the development of PTDM. The combinations of immunosuppressive treatment tacrolimus (TAC), cyclosporine A (CYC), and everolimus (EVL), steroids increase the incidence of PTDM significantly. Insulin is the most effective treatment for PTDM in the early transplant period; however, oral anti-diabetic medications look promising. Further clinical trials are required to determine the optimum treatment method for reducing the occurrence of PTDM and treating the existing condition with novel anti-hyperglycemic medications.

## Introduction and background

In 1954, Richard and Ronald Herrick underwent the first successful solid organ transplantation (SOT) and set an ideal solution for end-stage kidney disease (ESKD) patients worldwide. However, SOT has been associated with complications [1,2]. It was not until 2003 that guidelines established diagnosis and management recommendations for new-onset diabetes after transplantation (NODAT) [3]. However, in 2013, after an International Consensus meeting, this term was changed to post-transplant diabetes mellitus (PTDM). The term NODAT was ambiguous as it implied the exclusion of diabetes mellitus (DM) before transplantation and developed only after transplantation when there was no information about DM tests being done before the transplantation [2].

PTDM is a significant complication in renal transplant patients with no known history of DM pre-transplant [4,5]. Although PTDM shares a similarity in pathogenesis with Type 2 DM, it has other different mechanisms by which it can cause hyperglycemia [6]. Transient hyperglycemia (TH) does occur in about 80% of patients immediately after surgery due to perioperative stress, but this should not be misdiagnosed as PTDM. Nevertheless, patients with TH should be carefully monitored as they may develop PTDM in the future [7].

Incidence

In the United States, DM has developed in 2% to 50% of patients who did not have it before the transplant [6]. Under the current immunosuppressive regimen of tacrolimus (TAC), mycophenolic acid, and steroids, the incidence of PTDM is estimated to be between 20% and 30% [4]. According to the United States Renal Data System (USRDS) data from 1996-2000, PTDM occurs in 16% and 24% of cases at one and three years, respectively, following kidney transplantation (KT) [8]. Historically, the lack of consistent diagnostic criteria is attributed to variations in the prevalence of DM. After the guidelines criteria provided in 2003, the prevalence of DM is 10-30% [6].

Diagnosis

Timely screening of people at risk can help prevent PTDM [9]. The diagnostic criteria are (a) fasting glucose ≥ 126mg/dL (7mmol/L) in more than one instance, (b) random glucose ≥ 200 mg/dL (11.1mmol/L) with polyphagia, polyuria, polydipsia, (c) two-hour glucose after a 75g oral glucose tolerance test (OGTT) ≥ 200mg/dL (11.1mmol/L), and (d) hemoglobin A1C (HbA1c) ≥ 6.5%. The American Diabetes Association (ADA) recommends that if a patient's findings from two independent tests disagree, the test result above the diagnostic cut-off point should be redone, with the possibility of HbA1C assay interference taken into account. Thus, if a patient passes the HbA1C diabetes criterion but not the fasting plasma glucose criterion, that person should be confirmed as a diabetic [10]. Immunosuppressants and steroids can cause DM right after transplantation. As a result, capillary blood glucose monitoring should be performed after lunch and dinner to screen for hyperglycemia immediately post-transplant. HbA1c testing should be done three months after the transplant since blood loss, iron shortage, and blood transfusion can impact HbA1c levels in the perioperative [1]. 

The most modifiable risk factor for the development of PTDM is immunosuppression, however, to balance this risk against the risk of rejection, a risk vs. benefit analysis is needed [11]. Glucose-lowering medications have been shown to improve PTDM, but extensive research is needed to determine their safety and effectiveness [12,13]. In this study, we will be comparing the different antidiabetic drugs to manage PTDM.

## Review

Method

Search Strategy

This systematic review was designed using the Preferred Reporting Items for Systematic Reviews and Meta-Analyses (PRISMA) guidelines issued in 2020 [14]. The PubMed database was explored by employing the use of appropriate keywords and boolean operators. PubMed Central (PMC), Multidisciplinary Digital Publishing Unit (MDPI), and Elsevier databases were also explored. The terms and expressions were swapped with medical subject headings (MeSH) terms to precisely unearth all the apposite articles.

Keywords and key terms used include diabetes mellitus (DM), type 2 diabetes mellitus, hyperglycemia, increased blood glucose levels, tacrolimus (TAC), a calcineurin inhibitor, immunosuppressive drugs, mycophenolic acid, disease-modifying anti-rheumatic drugs (DMARD), immunosuppressive drugs, cyclosporine A (CYC), steroids, kidney transplantation, renal transplant, post renal transplantation, and immunocompromised patients.

As an application of the MeSH strategy, some expressions involving boolean operators are as follows: "Diabetes Mellitus, Type 2/complications"[Majr] OR "Diabetes Mellitus, Type 2/etiology"[Majr] OR "Diabetes Mellitus, Type 2/physiology"[Majr] OR "Diabetes Mellitus, Type 2/physiopathology"[Majr] ) OR Hyperglycemia OR increased blood glucose levels AND "Tacrolimus"[Majr] OR Calcineurin inhibitor OR Immunosuppressive drugs AND "Mycophenolic Acid"[Majr] OR DMARD OR Immunosuppressive drugs AND "Kidney Transplantation"[Majr] OR Renal transplant OR Post-Renal Transplantation OR immunocompromised patients. A PubMed collection of 213412 articles was generated. We also had a small collection of 35 publications from PMC, MDPI, and Elsevier.

Eligibility Criteria 

We applied inclusion and exclusion criteria to this collection as follows: full text, associated data, clinical trials, meta-analysis, randomized control trial (RCT), review, systematic reviews, last five-year studies, humans only, English, Medline, age group 19 and above. This left us with an endnote collection of 1020 PubMed articles. Further title screening thinned the search results to 27, and abstract screening further trimmed it to nine. A similar screening when applied to the other three databases pruned the search result of 35 publications to 26.

**Figure 1:**
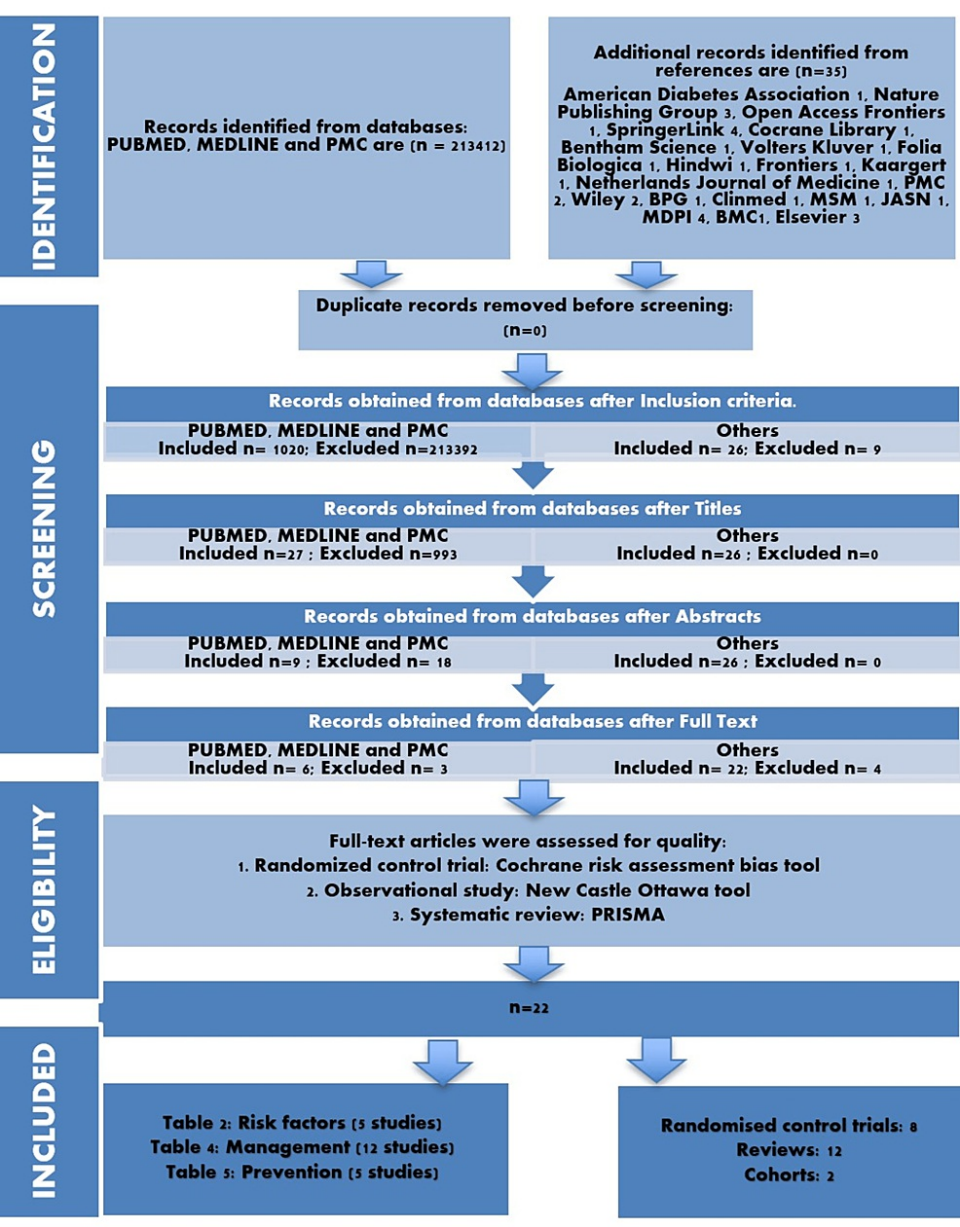
The Preferred Reporting Items for Systematic Reviews and Meta-Analyses (PRISMA) 2020 guidelines PMC- PubMed Central, BPG- Baishideng Publishing Group, JASN-Journal of the American Society of Nephrology, MDPI- Multidisciplinary Digital Publishing Institute, MSM-Medical Science Monitor, BMC- Biomed Central

Figure 1 demonstrates the PRISMA flowchart [14].

Results

By exploring the databases, we discovered a total of 213447 papers. 1046 records were filtered using inclusion-exclusion criteria, and 116 studies were evaluated based on the title and suitable abstract and full texts. We also deleted duplicate studies. After setting a 70% bar, we evaluated quality papers, and only 22 qualified after using the quality assessment tools. 

We employed the following methods: clinical trials = Cochrane Risk Bias Assessment tool, observational studies = Newcastle Ottawa, Appraisal Tool for Cross-sectional Studies (AXIS), a systematic review and meta-analysis = Assessment of Multiple Systematic Reviews (AMSTAR), literature review articles = Scale for the Assessment of Narrative Review Articles (SANRA) [15-18].

A total of 1333 patients from eight randomized control trials reported the advantages of adding CYC, using low-dosage TAC, and using metformin, vildagliptin, and sodium-glucose co-transporter 2 (SGLT2) inhibitors for the management of PTDM. An observational cohort study of 450 participants revealed significant risk factors for PTDM. A retrospective cohort analysis of 40108 individuals documented the use of immunosuppressant drugs and found them to be a risk factor for diabetes mellitus.

Discussion

The key objectives of this discussion include the multiple risk factors that cause PTDM, the benefits and risks of current glucose-lowering drugs for diabetes, and prevention approaches.

Risk Factors Responsible for PTDM

PTDM does not occur in all kidney transplant recipients. Risk factors for PTDM have been classified as modifiable and non-modifiable. Alternatively, "pre-transplant," "peri-transplant," and "post-transplant" terms are also used [7]. These are described in Table 1. 

**Table 1:** Risk factors responsible for post-transplantation diabetes mellitus

	Pre-transplant	Peri-transplant	Post-transplant
Modifiable	Pre-diabetes	Renal allograft	Cytomegalovirus (CMV)
			Hepatitis C virus (HCV)
	Obesity		Immunosuppressant drugs
			Hypomagnesemia
Non-modifiable	Age above 40		
	Genetics		
	Family history of diabetes mellitus		
	Polycystic kidney disease (PCKD)		
	Ethnicity		

Non-modifiable factors: After age>40, the likelihood of getting PTDM increases as beta-cell function reduces, leading to insulin resistance [9]. The incidence of PTDM follows a biphasic pattern, with the first peak being in the first few months after transplantation and the second peak being over the next two to three years [19]. Patients with numerous predisposing single nucleotide polymorphisms (SNP) like transcription factor 7-like 2 (TCF7L2), protein-encoding gene potassium inwardly-rectifying channel subfamily J member 11 (KCNJ11), lipid gated inward rectifier potassium ion channel (Kir6.2) which is a major subunit of the adenosine triphosphate (ATP)-sensitive potassium channel, interleukin (IL), and nuclear factor of activated T-Cells isoform c4 (NFATc4) are more likely to develop PTDM. Polymorphisms in the hepatocyte nuclear factor-4-alpha (HNF-4A) and insulin receptor substrate one genes have been linked to the development of PTDM in Hispanic renal allograft recipients [2]. However, a recent cohort study shows that the human leukocyte antigen (HLA) genotype had no role in PTDM or is still unknown [9]. In a cohort study, a family history of DM was identified as a risk factor for PTDM. Also, a meta-analysis of patients with autosomal dominant polycystic kidney disease indicated that those with the disease have a higher risk of developing PTDM than those who do not. According to a comparative case-control study, people of South Asian ancestry had a higher chance of PTDM than white people [19].

Modifiable factors: Body mass index (BMI)>30, metabolic syndrome, prediabetes, and occult diabetes are some of the significant pre-transplant risk factors. Immunosuppressant drugs like corticosteroids, TAC, CYC, and Everolimus (EVL) contribute significantly to the post-transplant risk. Belatacept is a more recent immunosuppressant. In a retrospective analysis, Belatacept with TAC and Belatacept alone were found to have a considerably reduced incidence of PTDM than TAC alone [7].

Cytomegalovirus (CMV): CMV is an opportunistic pathogen that thrives in immunosuppressed patients. CMV infection can cause diabetes by the death of pancreatic beta cells or the generation of pro-inflammatory cytokines, and it can independently develop PTDM. The relative risk of PTDM in CMV-positive individuals is 1.94 times higher than in CMV-negative patients, according to a meta-analysis that included data from 1389 renal transplant recipients [2].CMV infection quadruples the risk of PTDM in the first three months after KT [8]. The protective role of maximum antiviral drug use in the development of PTDM is still to be investigated, although such research would undoubtedly be beneficial in the future [19].

Hepatitis C virus (HCV): HCV causes DM by decreasing hepatic glucose absorption and glycogenesis. According to a study, it is responsible for increasing insulin resistance by 62%. However, it has no role in beta-cell activity [8].

Hypomagnesemia: Magnesium helps with the improvement of insulin sensitivity and glycemia in Type 2 DM patients. KT patients on CYC and TAC have low plasma magnesium levels due to urinary loss of magnesium. Thus, hypomagnesemia results in PTDM. However, treatment with oral magnesium has been ineffective so far [19].

Over three years, a cohort study on 450 receipts of both living and deceased donors was studied for PTDM risk factors. Both multivariate and univariate analysis showed that pre-transplant hyperglycemia and BMI >25 are pre-transplant variables associated with PTDM, while post-transplant transient hyperglycemia, acute rejection, calcium channel blockers, triglyceride/high-density lipoprotein (TG/HDL) ratio >3.5, and tacrolimus trough levels are post-transplant variables to cause PTDM. Identifying these risk factors aids in patient risk assessment and developing effective risk-reduction strategies for PTDM in the KT setting [9].

​Table 2, shown below, summarizes studies related to the risk factors of PTDM.

**Table 2:** Risk factors of PTDM PTDM- Post-transplantation diabetes mellitus

Study	Author	Year	Type of Study	Patient	Purpose of the study	Results	Conclusion
1	de Lucena et al. [9]	2020	Observational Cohort Study	450	Major risk factors for the development of PTDM are modifiable.	While recipient age, pre-transplant hyperglycemia, and BMI >25 are modifiable risk factors before transplant, acute rejection, triglyceride level, and tacrolimus level are modifiable risk factors after transplant.	In order to develop preventive strategies identification of modifiable risk factors as early as possible is important.
2	Klangjareonchai et al. [8]	2021	Review		Latest drugs used in the management of PTDM.	Results show that prevention of the risk factors of PTDM is equally important in addition to pharmacologic management.	There is little evidence available for clinicians to evaluate diabetes treatments.
3	Rodríguez-Rodríguez et al. [7]	2021	Review		The most recent research on PTDM therapy is reviewed.	Metabolic syndrome and insulin resistance are the pretransplant risk factors causing beta cell dysfunction.	According to the conclusions of this study, both PTDM and pre-diabetes mellitus are correlated to renal and cardiovascular problems.
4	Ahmed et al. [2]	2020	Review		This article offers a comprehensive study of PTDM's pathophysiology, diagnostic criteria, and management in the context of modern research. Drug safety and side effects are also highlighted.	Early screening and preventive approaches can help maintain pretransplant, peri-transplant, and post-transplant risk factors under control.	Identification of the risk factors for PTDM and using preventive strategies. Although the treatment plan is individualized
5	Jenssen et al. [19]	2019	Review		The purpose of this study is to review risk factors, pathogeneses, and management of PTDM.	This article's results suggest that insulin is beneficial for managing PTDM in the first month after transplantation, but that from the second month through long-term maintenance, dietary changes, anti-diabetic medications, immunosuppressants, and steroids are required.	The research concluded that lifestyle interventions should begin early, adequate immunosuppressive regimens should be employed, and further studies on antidiabetic drugs should be undertaken.

Management of PTDM 

As we know that immunosuppressants constitute a significant risk factor for PTDM, few studies have provided details about their incidence of causing PTDM, which we will be discussing further. Table 3 describes the mechanism of action of each drug.

**Table 3:** Mechanism of action of immunosuppressive drugs

	Decrease insulin secretion	Increase beta-cell apoptosis	Increase glucagon secretion	Increase glucose production	Decrease glucose uptake	Decrease incretin effect	Increased appetite
Steroids	✓	✓	✓	✓	✓	✓	✓
Cyclosporine	✓	✓					
Tacrolimus	✓	✓					
Sirolimus	✓	✓			✓		

Hypoglycemic drugs should be administered to patients with PTDM after they have passed safety and efficacy testing for organ function, immunosuppressive drug interactions, and drug-drug interactions. Both immunosuppressants and medications for decreasing blood sugar have been the subject of extensive, lengthy investigations [20].

An RCT studied the combination of EVL and low-dose TAC. The mammalian target of rapamycin (mTOR) inhibitor EVL was identified as an alternative immunosuppressant because of its reno-protective effect in the early stages. The EVL group showed encouraging results, with the estimated glomerular filtration rate (eGFR) improving at nine and 12 months compared to baseline with the standard-dose TAC group. However, EVL is diabetogenic, and switching from TAC to EVL in the early postoperative phase has increased the incidence of PTDM and slowed wound healing [21].

Two RCTs were conducted on renal transplant recipients. One is the ZEUS trial in which recipients were switched to EVL at 4.5 months or continued on CYC. Another trial is called HERAKLES in which recipients were switched to EVL at three months or converted to EVL with reduced-exposure CYC. The incidence of PTDM, random blood sugar, and eGFR were similar in both groups, with no appreciable changes. This means that there is no change in the severity of PTDM with the early switch to an EVL-based regimen [22].

An RCT including 87 patients reduced the use of oral anti-hyperglycemic agents and lowered HbA1C levels after switching from TAC to CYC by the end of a year [4].

This individualized approach for the management of PTDM depends on factors like ABCDE: A = Allograft function and adverse drug events, B = Body weight, C = Comorbidities, D = Drug-drug interactions, and E = Expenses.

Insulin: Initial management of PTDM includes basal isophane insulin. According to a 2018 RCT study, continuous subcutaneous insulin lispro infusion (CSII) treatment was a safer alternative to the standard treatment of insulin isophane. However, its role in the development of PTDM is yet to be studied [23].

Metformin: In a randomized, double-blind, placebo-controlled trial participants were studied on how metformin altered PTDM risk variables. According to the survey, adenosine 5′-monophosphate (AMP)-activated protein kinase (AMPK) activation can improve chronic metabolic disease risk factors, and metformin, a widely used oral hyperglycemic drug, operates on AMPK. In one study, metformin induces lactic acidosis, especially in those with an eGFR of less than 30mL/min; however, it is safe in people with an eGFR of 30-60mL/min [24]. Transdiab (Transplantation and Diabetes) is a randomized controlled experiment. There was no difference in weight gain, lipid profile, or efficacy between the control and metformin groups. However, in the metformin group, there was a trend of reduced weight gain and more patients returning to normal oral glucose tolerance test (OGTT) after 12 months. However, the study had a small sample size [3].

Glucagon-like peptide one receptor agonist (GLP-1): GLP-1s reduce hepatic glucose synthesis, release glucagon after meals, slow stomach emptying, and decrease central hunger. The effects of dulaglutide and liraglutide are similar. In contrast to liraglutide, dulaglutide has a longer duration of action, resulting in a reduced daily insulin need. Semiglutide helps in weight loss. GLP-1 agonists have cardioprotective and reno-protective effects. The side effect of GLP-1 agonists in KT patients is gastrointestinal intolerance, which aggravates in combination with mycophenolate [20].

Dipeptidyl peptidase-4 (DPP-4) inhibitors: DPP-4 inhibitors inhibit the breakdown of GLP-1, increase new beta cell formation, and thus improve blood glucose [20]. Kidneys usually excrete gliptins except for linagliptin and gemigliptin, which are excreted by bile. The advantages of linagliptin are it lowers the basal-bolus insulin demand in the early KT period, decreases HbA1C compared to other gliptins, and does not need renal dose adjustment [8]. In an RCT, vidalagliptin decreased the two-hour postprandial glucose compared to placebo. Thus, it helped reduce hyperglycemia-associated glucose and cardiovascular risks [25].

SGLT2 inhibitors: SGLT2 inhibitors lower blood glucose levels by blocking SGLT2 receptors, thereby decreasing glucose and sodium reabsorption in the proximal convoluted tubules and increasing glucose excretion in the urine. This aids in glycemic management promotes natriuresis and energy loss, and so aids in the reduction of obesity, cardiovascular consequences, and renal function, all of which are accomplished without the usage of insulin. Hepatic metabolism is the primary route of drug clearance for SGLT2 inhibitors [8]. Treatment with SGLT2 inhibitors in type 2 DM patients has been found to slow the course of chronic kidney disease (CKD) and adverse renal events progression, as shown in five clinical trials namely EMPA-REG, CANVAS, DECLARE, CREDENCE, and DAPA-CKD [20]. The most common risk factors associated with flozins are urinary tract infections due to increased glucose in the urine, and volume depletion due to natriuresis. The patient must avoid such occurrences in patients with recurrent urinary tract infections and severe dehydration. Empagliflozin appears to offer significant protection against major renal and cardiovascular consequences, according to EMPA-REG research. Empagliflozin was demonstrated in an experimental in vitro model to reduce tacrolimus-induced hyperglycemia while raising plasma insulin levels. In the CANVAS research, participants who were randomly assigned to the canagliflozin group experienced a lower incidence of cardiovascular events. The study also revealed that canagliflozin-assigned subjects were less likely to have end-stage renal disease, a decline in eGFR, or albuminuria progression [11]. According to an RCT trial results, individuals with bilateral nephrectomies showed decreased de novo glucose production for increased glucose loss caused by SGLT2 inhibitors indicating that the hepatic renal axis is deranged due to lack of efferent nerve stimulation [26].

Current research shows that islet cell transplantation is safe and improves glycemic control in Type 1 DM patients who have undergone KT. In the future, stem cells may minimize immunogenicity and reduce the need for continuous immunosuppression [6]. 

Table 4, shown below, summarizes studies related to the management of PTDM.

**Table 4:** Mangement of PTDM RCT- randomized control trial, SGLT2- sodium-glucose co-transporter 2, EVL- everolimus, TAC- tacrolimus, CYC- cyclosporine, NODAT- new-onset diabetes after transplantation, GFR-glomerular filtration rate, PTDM- post-transplantation diabetes mellitus, DPP4- dipeptidyl peptidase-4, GLP-1- glucagon-like peptide, LDL- low-density lipoprotein, CSII- continuous subcutaneous insulin lispro infusion, HbA1C- glycosylated hemoglobin

Study	Author	Year	Type of Study	Patients	Purpose of the study	Results	Conclusion
1	Yin et al. [24]	2021	RCT	105	The goal of this trial is to see if metformin and SGLT2 inhibitors improve metabolic profiles in kidney transplant patients.	The ratio of visceral to subcutaneous fat, lipid metabolism, graft function, and inflammatory indicators all influence the outcome.	The emergence of metabolic diseases is a long-term process influenced by a variety of factors, but because this study was only done for a year, it will only look at short-term consequences.
2	Kim et al. [21]	2021	RCT	77	The aim of the study is to use a combination of EVL and low-dose TAC for the development of NODAT.	The findings after three months were similar; however, EVL improved the GFR after 12 months while TAC increased insulin resistance.	For maintenance treatment, this combination is useful.
3	Daniele et al. [26]	2020	RCT	20	This study aims to see the effect of endogenous glucose production in bilaterally nephrectomized patients on SGLT2 inhibitors.	The liver normally compensates for urinary glucose loss caused by SGLT2 inhibitors by increasing hepatic glucose production but in bilateral nephrectomized individuals, this compensation is reduced.	A significant aspect of the renal-hepatic axis has been found as a result of this research.
4	Gaiffe et al. [25]	2019	RCT	84	This study aims to use Vidalagliptin for preventing PTDM immediately after renal transplantation for two months.	The outcome of this study is determined by the patient's overall health and fasting glucose level more than seven or HbA1C after one year.	This study concluded that using vidalagliptin may reduce PTDM, but the study also states that it is more useful in non-transplant patients with other comorbidities.
5	Alnasrallah et al. [3]	2019	RCT	78	Managment of PTDM with Metformin.	The results in the control group and the group using metformin were similar.	In order to know the efficacy of metformin a trial on more number of patients is required.
6	Wissing et al. [4]	2018	RCT	87	The aim of the study is to switch from TAC to CYC manage PTDM.	CYC proved beneficial by improving the HbA1C and reducing the use of anti-diabetic medication at the end of 12 months.	TAC to CYC conversion provided beneficial outcomes, possibly by reversing diabetes in the first year, however, it has been attributed to a rise in reports of infectious diseases and an increase in LDL levels.
7	Sommerer et al. [22]	2018	RCT	797	The aim of the study is to see the development of diabetes mellitus in patients on converting from CYC to EVL.	There was no significant difference in Zeus and Herackles study group test results.	This study concludes that there is no change in the occurrence of PTDM with early conversion of CYC to EVL.
8	Werzowa et al. [23]	2018	RCT	84	This trial is about using subcutaneous insulin lispro infusion (CSII) treatment in the first week of transplant.	The use of CSII showed improvement in blood glucose for a brief period than basal isophane insulin and control group.	The prevention of PTDM is further dependent on the outcomes of current clinical trials on insulin.
9	Montero et al. [20]	2022	Review		To study the new drugs in the management of post-transplant diabetes mellitus.	According to the most recent guidelines, additional drugs including GLP-1 agonists, DPP4 inhibitors, and SGLT2 inhibitors are currently being added to metformin for the treatment of PTDM.	It is vital to understand the mechanism of action and benefits of new drugs in the renal transplant patient group.
10	Schwarzenbach et al. [11]	2021	Review		Role of sodium-glucose cotransporter 2 inhibitors in transplant patients.	SGLT2 inhibitors improve cardiovascular health and help in weight reduction in transplant patients while causing minimal urinary tract infections.	The information regarding SGLT2 inhibitors in renal transplant patients is limited and more prospective trials are needed.
11	Klangjareonchai et al. [8]	2021	Review		Latest drugs used in the management of PTDM.	This study's results show that prevention of the risk factors of PTDM is equally important in addition to pharmacologic management.	There is little evidence available for clinicians to evaluate diabetes treatments.
12	Martinez Cantarin et al. [6]	2021	Review		This is a review of the pathogenesis of PTDM.	Beta cell toxicity by immunosuppressants leads to insulin sensitivity and decreased insulin secretion. Also, the pancreatic gut incretin axis changes lead to PTDM.	Diabetes current treatment has gained significance in recent years, although its value in improving results in PTDM management is unknown.

Prevention of PTDM

Before transplantation, all candidates should have their diabetes risk assessed thoroughly [10]. Chakkera et al. observed six indicators for identifying high-risk populations for focused prevention of PTDM: age, planned corticosteroid therapy post-transplant, gout medicine prescription, BMI, fasting glucose and triglycerides, and a family history of type 2 diabetes. However, two parameters, planned corticosteroid therapy after transplant and prescription for gout medicine, were excluded since they were considered biased due to clinician prescription subjectivity. Furthermore, fasting glucose and triglycerides cannot be used as selection criteria either, as they both need a 12-hour fast, which is not advisable for individuals who have been chosen for a kidney transplant [25].

Exercise: Daily routine exercises are a straightforward and effective way to prevent PTDM. Physical activity initiates 5-alpha-AMPKs, which promote glucose absorption and fatty acid oxidation in the body. Translocation of glucose transporter (GLUT4) from intracellular storage sites to the cellular membrane is essential for exercise-induced glucose absorption. Physical activity improves muscle glucose uptake, a critical component of PTDM prophylaxis [27]. EXPRED (Exercise and Prediabetes after Transplantation) is one of the few studies that look at the ability of exercise to reverse prediabetes in patients who have been on the transplant list for at least 12 months. The findings could indicate that exercise is effective in preventing late PTDM. However, no research has looked into the effect of exercise or other interventions on the reversibility of the risk profile for PTDM in patients on the transplant waiting list, which is an issue worth exploring [7].

Diet: The Mediterranean style diet emphasizes whole grains, legumes, fruit, vegetables, olive oil, and fish while limiting dairy and meat consumption which are rich in high antioxidants, fiber, magnesium, and unsaturated fatty acids and are thought to increase insulin sensitivity and pancreatic beta-cell function, as well as reduce inflammation and endothelial dysfunctions. All KT patients should take a high-energy, moderate protein, low-fat diet and food containing three essential minerals: phosphorus, magnesium, and vitamin D [8].

Basal insulin: According to the TIP study (Treat-to-target trial of basal insulin to oral therapy in PTDM), recipients who were given basal insulin which is the isophane insulin for evening blood glucose > 140 mg/dL during the first three weeks after KT had significantly lower HbA1C levels than those who were given standard-of-care control which is antihyperglycemic agents for blood glucose > 180-250 mg/dL at three months and later. As a result, treating individuals with basal insulin reduced the likelihood of DM [8].

Bariatric surgery: The primary goal of bariatric surgery is to improve accessibility to transplantation and pretransplantation health status for people whose obesity prevents them from receiving KT. Additionally, sleeve gastrectomy reduces diabetes prevalence in CKD patients. A study found that 20 renal transplant recipients who underwent pretransplant laparoscopic sleeve gastrectomy had a lower incidence of PTDM than 40 controls with matched age, gender, and BMI [27].

Table 5, shown below, summarizes studies related to the prevention of PTDM.

**Table 5:** Prevention of PTDM PTDM- post-transplantation diabetes mellitus, RCT- randomized control trial, HbA1C- glycosylated hemoglobin

Study	Author	Year	Type of Study	Patients	Purpose of the study	Results	Conclusion
1	Gaiffe et al. [25]	2019	RCT	84	This study aims to use Vidalagliptin for preventing PTDM immediately after renal transplantation for two months.	The outcome of this study is determined by the patient's overall health and fasting glucose level of more than seven or HbA1C after one year.	This study concluded that using vidalagliptin may reduce PTDM, but the study also states that it is more useful in non-transplant patients with other comorbidities.
2	Ducloux et al. [27]	2022	Review		Not all, but many PTDM risk factors are modifiable, therefore it is critical to understand the preventative measures discussed in this study.	The different preventive measures are diet, bariatric surgery, microbiota, physical activity, and drugs.	More research studies on PTDM prevention techniques are required.
3	Klangjareonchai et al. [8]	2021	Review		Latest drugs used in the management of PTDM.	This study's results show that prevention of the risk factors of PTDM is equally important in addition to pharmacologic management.	There is little evidence available for clinicians to evaluate diabetes treatments.
4	Ponticelli et al. [10]	2021	Review		The purpose of this study is to review new-onset diabetes mellitus in renal transplant patients.	It showed that new-onset diabetes mellitus can lead to both microvascular and macrovascular complications.	This study concludes that new-onset diabetes mellitus is the most frequent complication of renal transplantation and immunosuppressant medications are the most common cause.
5	Rodríguez-Rodríguez et al. [7]	2021	Review		The most recent research on PTDM therapy is reviewed.	Metabolic syndrome and insulin resistance are the pretransplant risk factors causing beta cell dysfunction.	According to the conclusions of the study, both PTDM and pre-diabetes mellitus are correlated to renal and cardiovascular problems.

## Conclusions

One can successfully treat end-stage renal disease with kidney transplantation. A significant complication associated with this is the development of PTDM, which can affect graft survival. Identifying the risk factors for DM and monitoring the OGTT and HbA1C values is crucial as an early prevention strategy. Once we establish PTDM, management is a multidisciplinary approach that includes lifestyle changes, proper immunosuppressive regimens, early insulin initiation, and oral anti-diabetic drugs. However, there is still a need for further research on anti-hyperglycemic medications, focusing on including a larger population in the trial and its effects on kidney, cardiovascular, and graft health.
